# Effect of Global Climate Change-Related Factors on COPD Morbidity

**Published:** 2017

**Authors:** Hasan Bayram

**Affiliations:** 1 University of Gaziantep, Turkey; 2 Koc University, Istanbul, Turkey.

Epidemiological studies suggest an association between particulate air pollution and respiratory and morbidity due to chronic obstructive pulmonary disease (COPD). However, the role of factor-related global climate change including meteorological variables and desert dust storms has not been adequately addressed. Recently, we investigated the association between daily temperatures and desert dust storms, particulate matter (PM) levels, and emergency room visits and hospitalization due to COPD. Records of emergency room visits and hospitalization due to COPD were recruited from the registry of Gaziantep University Research and Training Hospital, whereas meteorological data, PM levels and records for dust storms were obtained from local monitoring stations and satellite pictures. A generalized additive regression model was built to investigate effects of variables studied on COPD admission and hospitalization by adjusting possible confounding factors. Of the meteorological parameters, maximum daily temperature inversely associated with both emergency room visits and hospitalization due to COPD at lag 0. Although dust storms increased the risk for emergency room visits due to COPD at lag 0, there was no significant effects of dust storms on hospitalization. Furthermore, there was a positive association between PM levels and emergency room visits at lag 0, and this was significant both at lag 3 and lag 4. PM levels also increased the risk for hospitalization in COPD patients. Our findings suggest that low temperatures, desert dust storms, PM levels may have an impact on COPD morbidity.

